# Immunobiotic *Ligilactobacillus salivarius* FFIG58 Confers Long-Term Protection against *Streptococcus pneumoniae*

**DOI:** 10.3390/ijms242115773

**Published:** 2023-10-30

**Authors:** Mariano Elean, Fernanda Raya Tonetti, Kohtaro Fukuyama, Luciano Arellano-Arriagada, Fu Namai, Yoshihito Suda, Nadia Gobbato, Keita Nishiyama, Julio Villena, Haruki Kitazawa

**Affiliations:** 1 Laboratory of Immunobiotechnology, Reference Centre for Lactobacilli (CERELA-CONICET), Tucuman 4000, Argentina; melean@cerela.org.ar (M.E.); frayatonetti@gmail.com (F.R.T.); luciarellano1996@gmail.com (L.A.-A.); 2 Food and Feed Immunology Group, Laboratory of Animal Food Function, Graduate School of Agricultural Science, Tohoku University, Sendai 981-8555, Japan; kotaro.fukuyama.p8@dc.tohoku.ac.jp (K.F.); fu.namai.a3@tohoku.ac.jp (F.N.); keita.nishiyama.a6@tohoku.ac.jp (K.N.); 3 Livestock Immunology Unit, International Education and Research Center for Food and Agricultural Immunology (CFAI), Graduate School of Agricultural Science, Tohoku University, Sendai 981-8555, Japan; 4 Department of Food, Agriculture and Environment, Miyagi University, Sendai 980-8572, Japan; suda@myu.ac.jp; 5 Laboratory of Immunology, Microbiology Institute, Faculty of Biochemistry, Chemistry and Pharmacy, National University of Tucuman, Tucuman 4000, Argentina; nadiagobbato@hotmail.com

**Keywords:** *Ligilactobacillus salivarius* FFIG58, respiratory superinfection, *Streptococcus pneumoniae*, poly(I:C), antiviral immunity, immunobiotics

## Abstract

Previously, we isolated potentially probiotic *Ligilactobacillus salivarius* strains from the intestines of wakame-fed pigs. The strains were characterized based on their ability to modulate the innate immune responses triggered by the activation of Toll-like receptor (TLR)-3 or TLR4 signaling pathways in intestinal mucosa. In this work, we aimed to evaluate whether nasally administered *L. salivarius* strains are capable of modulating the innate immune response in the respiratory tract and conferring long-term protection against the respiratory pathogen *Streptococcus pneumoniae*. Infant mice (3-weeks-old) were nasally primed with *L. salivarius* strains and then stimulated with the TLR3 agonist poly(I:C). Five or thirty days after the last poly(I:C) administration mice were infected with pneumococci. Among the strains evaluated, *L. salivarius* FFIG58 had a remarkable ability to enhance the protection against the secondary pneumococcal infection by modulating the respiratory immune response. *L. salivarius* FFIG58 improved the ability of alveolar macrophages to produce interleukin (IL)-6, interferon (IFN)-γ, IFN-β, tumor necrosis factor (TNF)-α, IL-27, chemokine C-C motif ligand 2 (CCL2), chemokine C-X-C motif ligand 2 (CXCL2), and CXCL10 in response to pneumococcal challenge. Furthermore, results showed that the nasal priming of infant mice with the FFIG58 strain protected the animals against secondary infection until 30 days after stimulation with poly(I:C), raising the possibility of using nasally administered immunobiotics to stimulate trained immunity in the respiratory tract.

## 1. Introduction

Lower respiratory tract infections are a major global concern because of their high morbidity and mortality in high-risk populations, causing more than 4 million deaths worldwide every year [1]. The respiratory syncytial virus (RSV) is the main causative agent of respiratory tract infections in children [2]. Studies have shown that before the age of two practically all children will be infected with this virus [3]. RSV infection is normally self-limited by the immune system in immunocompetent adults; however, children due to undeveloped immunity, the elderly, and immunocompromised individuals can developed a severe disease [2]. RSV is one of the leading causes of death from infections caused by a single pathogen in infants under 5 years of age, especially in undeveloped countries [4,5]. In addition to the complications associated with primary RSV infection, the virus can induce a TLR3-mediated inflammatory response in the lung causing the disruption of epithelial barriers and favoring secondary bacterial infections [6]. Among pathogens, *Streptococcus pneumoniae* is the most common bacteria identified as a cause of secondary respiratory infection [7,8]. If not controlled by the host, secondary infections by *S. pneumoniae* can cause pneumonia, sepsis, meningitis, and even death.

In recent years, the use of probiotic lactic acid bacteria (LAB) with immunomodulatory capacities (immunobiotics) has been proposed to increase immune defenses against bacterial or viral pathogens in the respiratory tract [9]. Different studies highlight the ability of live or heat-inactivated bacteria or their cell fractions to confer protection in the respiratory tract against pathogens such as RSV and *S. pneumoniae* [10]. Although the mechanism of action of probiotics on the immune system is not yet elucidated, current evidence suggests that these bacteria would be capable of interacting with pattern recognition receptors (PRRs) such as TLRs and nucleotide-binding oligomerization domain receptors (NODs) to stimulate innate immunity [11]. These improvements to innate immunity were initially thought to be short-term effects. However, it was seen that the stimulation of innate immunity can generate long-term effects due to metabolic reprogramming and DNA methylation changes [12]. This innate immune memory was recently named as “trained immunity” and defined as the ability of cells of the innate immune system to gain memory characteristics after transient stimulation, resulting in an enhanced response upon secondary challenge with the same or an unrelated stimulus [12]. Although knowledge of trained immunity has advanced remarkably in recent years, little is known about how probiotic bacteria can influence trained immunity.

We hypothesized that the use of immunobiotic beneficial microbes with the ability to modulate trained immunity in the respiratory tract could be of value in enhancing long-term protection against both bacterial and viral pathogens. In a previous study, we demonstrated that feeding pigs with the algae wakame (*Undaria pinnatifida*) not only improved intestinal health and conferred protection against pathogens but also modified microbiota composition, increasing the relative abundance of *Ligilactobacillus salivarius* [13]. We associated the beneficial effect of wakame with the higher abundance of *L. salivarius* species, and then carried out the isolation of potentially probiotic *L. salivarius* strains from the intestine of pigs fed with the wakame algae [13]. The *L. salivarius* strains isolated were characterized based on their ability to modulate the innate immune responses triggered by the activation of TLR3 or TLR4 signaling pathways in porcine intestinal epithelial (PIE) cells [13,14]. Based on these results, we selected five strains (*L. salivarius* FFIG35, FFIG56, FFIG58, FFIG79, and FFIG124) to further investigate their ability to confer protection in the respiratory tract of mice against viral and bacterial pathogens.

In this work, we aimed to evaluate whether nasally administered porcine *L. salivarius* strains are capable of modulating the innate immune response in the respiratory tract and conferring long-term protection against the respiratory pathogen *Streptococcus pneumoniae*. We evaluated the changes in the cytokine profile and lung damage in a murine model of superinfection that combined challenges with poly(I:C) and *S. pneumoniae*. Considering the preponderant role of alveolar macrophages (AMs) as the first line of defense in the lung, we studied the ability of the strains to modulate their response to challenges with poly(I:C) or pneumococci. In addition, we studied the duration of the protection induced by the lactobacilli strains.

## 2. Results

### 2.1. Effect of Porcine L. salivarius Strains against Secondary Pneumococcal Infection

**Figure 1:**
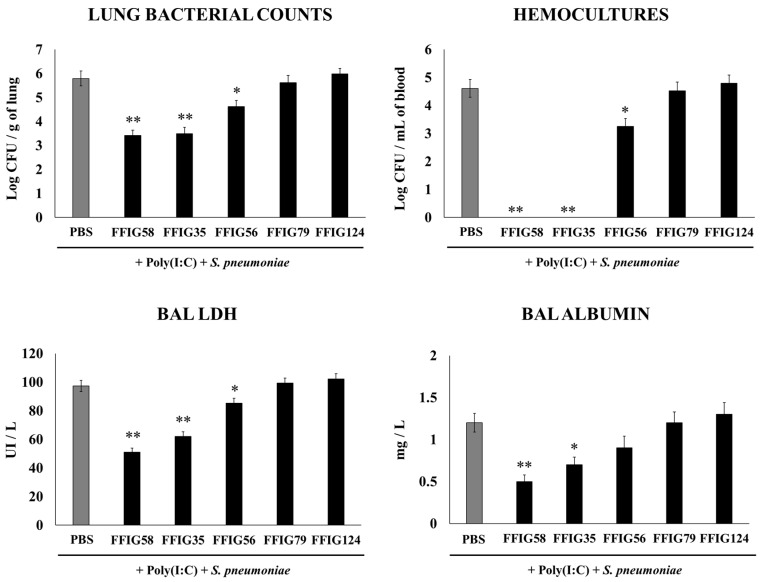
Effect of *L. salivarius* FFIG35, FFIG56, FFIG58, FFIG79, and FFIG124 on the resistance to secondary pneumococcal pneumonia after poly(I:C) treatment. Infant mice were nasally primed with the strains during two consecutive days, then stimulated with three once-daily doses of poly(I:C) and finally challenged with *S. pneumoniae* serotype 6B five days after the last administration of poly(I:C). Infant mice treated with PBS (vehicle), stimulated with poly(I:C), and then challenged with *S. pneumoniae* were used as controls. Lung bacterial cells counts, hemocultures, lactate dehydrogenase (LDH) activity, and albumin concentrations in bronchoalveolar lavages (BALs) were determined on day 2 post-pneumococcal-challenge. Asterisks indicate significant differences between lactobacilli-treated and control groups; * (*p* < 0.05) and ** (*p* < 0.01).

The capacity of *L. salivarius* strains isolated from the intestine of wakame-fed pigs to modulate the innate immune response in the respiratory tract was evaluated. We used a model of secondary *S. pneumoniae* infection induced after poly(I:C) stimulation [15]. Infant mice were nasally treated with the different *L. salivarius* strains, stimulated with poly(I:C), and then challenged with *S. pneumoniae*, as described in the Materials and Methods section. Bacterial cell counts in the lung and blood, and lung-injury-related biochemical markers were evaluated two days after the pneumococcal challenge (Figure 1).

The analysis of the pneumococcal counts in the lung and blood cultures revealed strain-dependent differences in the ability to improve protection against the secondary *S. pneumoniae* infection. The groups of mice treated with the FFIG35 and FFIG58 strains showed the lowest counts of pneumococci in the lung, and no colonies were detected in their hemocultures (Figure 1). In addition, the treatment with FFIG56 induced a significant decrease in both blood and lung bacterial counts, but its protective effectiveness was significantly lower than that observed for the strains FFIG35 and FFIG58. In contrast, the animals treated with the strains FFIG79 and FFIG128 had *S. pneumoniae* counts in lung and blood that were similar to the controls (Figure 1).

In order to study the inflammatory-mediated lung damage, LDH activity and albumin content were determined in BALs (Figure 1). The treatments with the FFIG58 and FFIG35 strains were the most effective in reducing lung tissue damage, as shown by the significantly lower levels of albumin content and LDH activity compared with the controls. The treatment with *L. salivarius* FFIG56 also reduced the levels of BAL LDH but the values were significantly higher than those observed in the FFIG58 and FFIG35 groups. The FFIG79 and FFIG124 strains were unable to reduce lung damage, and showed similar levels of LDH activity and albumin content to the control group (Figure 1).

### 2.2. Effect of Porcine L. salivarius Strains on BAL Cytokine Profiles in Response to Secondary Pneumococcal Infection

**Figure 2:**
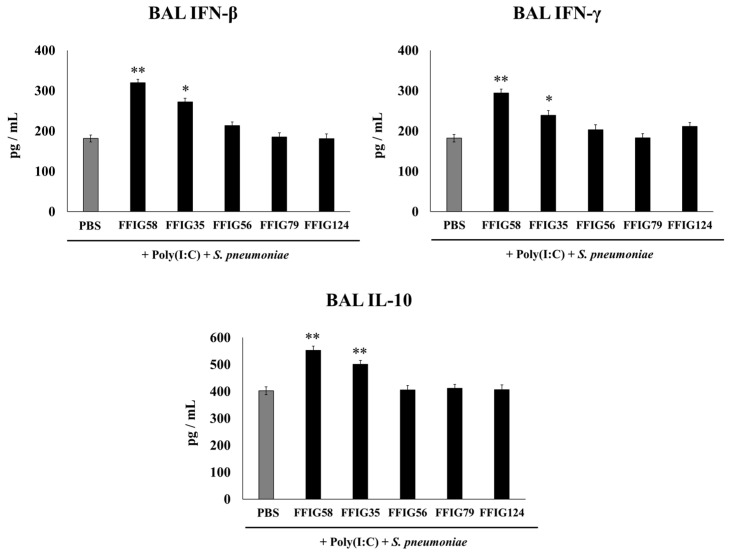
Effect of *L. salivarius* strains on the levels of interferons (IFN)-β and IFN-γ, and interleukin (IL)-10 in a secondary pneumococcal pneumonia model. Infant mice were nasally primed with FFIG35, FFIG56, FFIG58, FFIG79, or FFIG124 for two consecutive days, then stimulated with three once-daily doses of poly(I:C), and finally challenged with *S. pneumoniae* serotype 6B five days after the last administration of poly(I:C). Infant mice treated with PBS (vehicle), stimulated with poly(I:C), and then challenged with *S. pneumoniae* were used as controls. The levels of IFN-β, IFN-γ, and IL-10 in bronchoalveolar lavages (BALs) were determined on day 2 post-pneumococcal-challenge. Asterisks indicate significant differences between lactobacilli-treated and control groups; * (*p* < 0.05) and ** (*p* < 0.01).

Considering the critical role played by the cytokines in the resolution of lung infections, we next aimed to evaluate the impact of the different treatments on the cytokines produced in the respiratory tract in response to poly(I:C) and *S. pneumoniae* challenges. Thus, the levels of IFN-β, IFN-γ, and IL-10 were quantified in the BAL samples (Figure 2) taking into account that these cytokines are differentially regulated by nasally administered probiotic microorganisms [15,16,17]. The nasal priming with *L. salivarius* FFIG58 stood out as the most effective treatment to enhance the levels of the three cytokines tested. Similar results were obtained with the strain FFIG35 although the average values obtained were lower than those observed in the FFIG58 group. No significant differences were found between the control group and the groups receiving the strains FFIG56, FFIG79, or FFIG128 (Figure 2).

### 2.3. Effects of Porcine L. salivarius FFIG58 on Alveolar Macrophages in Response to Secondary Pneumococcal Infection

**Figure 3:**
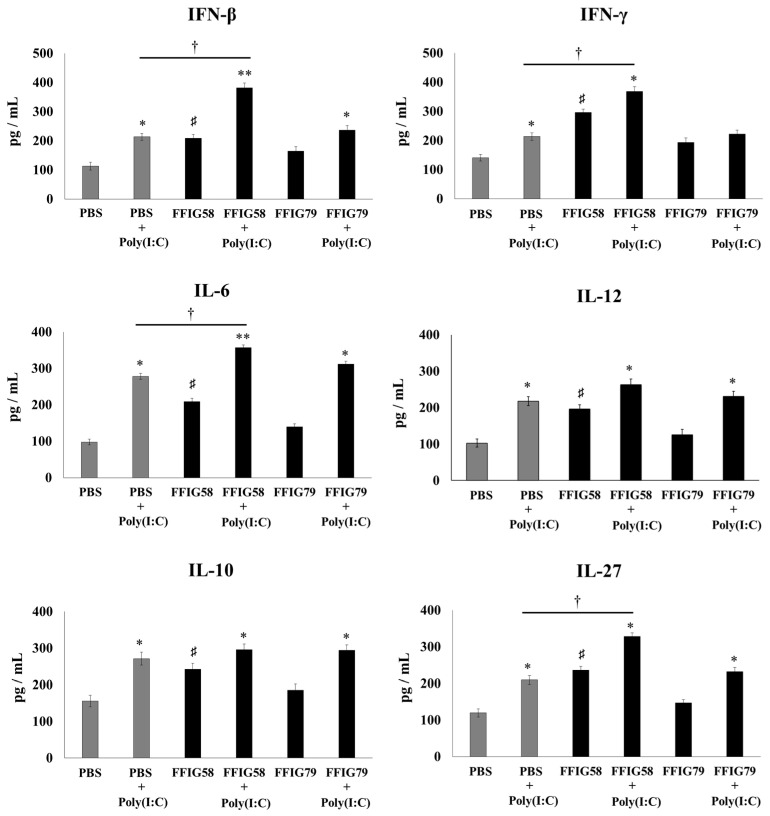
Effect of *L. salivarius* FFIG58 (positive strain) and FFIG79 (negative strain) on the production of interferons (IFN)-β and IFN-γ, and interleukins (IL)-6, IL-10, IL-12, and IL-27 by alveolar macrophages in response to poly(I:C) challenge. Infant mice were nasally primed with FFIG58 or FFIG79 for two consecutive days while control mice received PBS (vehicle). Alveolar macrophages were isolated from infant mice on the third day and challenged in vitro with poly(I:C). The levels of IFN-β, IFN-γ, IL-6, IL-10, IL-12, and IL-27 were evaluated in alveolar macrophages by enzyme-linked immunosorbent assay (ELISA) before (basal) and after (12 h) poly(I:C) challenge. The results represent data from three independent experiments. Asterisks indicate significant differences between basal and post-poly(I:C) challenge within the same group; * (*p* < 0.05) and ** (*p* < 0.01). Symbol (†) shows significant differences between the indicated groups; * (*p* < 0.05). Symbol (♯) indicates significant differences between lactobacilli-treated and PBS controls at the basal time point; * (*p* < 0.05).

**Figure 4:**
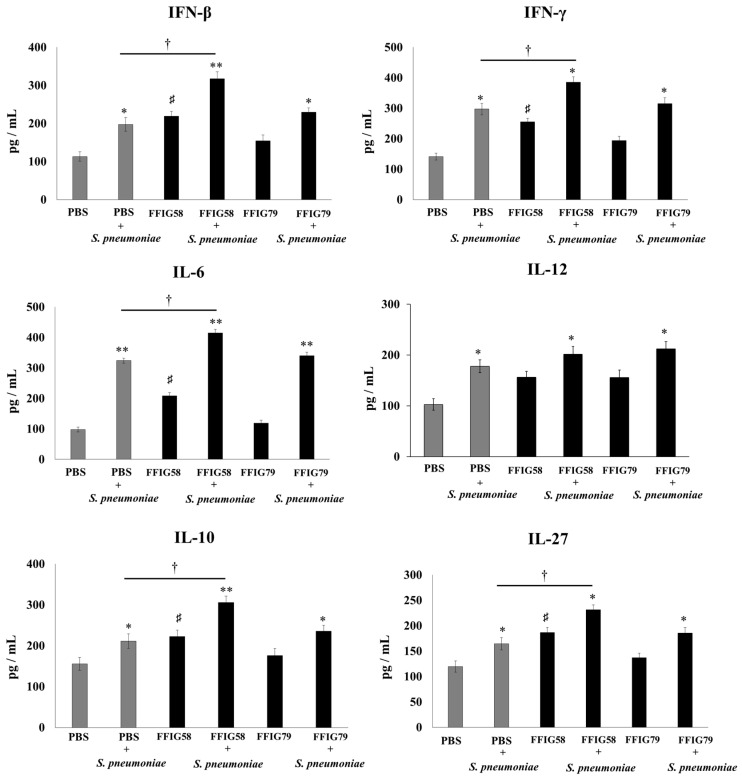
Effect of *L. salivarius* FFIG58 (positive strain) and FFIG79 (negative strain) on the production of interferons (IFN-β) and IFN-γ, and interleukins (IL)-6, IL-10, IL-12, and IL-27 by alveolar macrophages in response to *Streptococcus pneumoniae* challenge. Infant mice were nasally primed with FFIG58 or FFIG79 for two consecutive days while control mice received PBS (vehicle). Alveolar macrophages were isolated from infant mice on the third day and challenged in vitro with *S. pneumoniae*. The levels of IFN-β, IFN-γ, IL-6, IL-10, IL-12, and IL-27 were evaluated in alveolar macrophages by enzyme-linked immunosorbent assay (ELISA) before (basal) and after (12 h) *S. pneumoniae* challenge. The results represent data from three independent experiments. Asterisks indicate significant differences between basal and post-pneumococcal-challenge within the same group; * (*p* < 0.05) and ** (*p* < 0.01). Symbol (†) shows significant differences between the indicated groups; * (*p* < 0.05). Symbol (♯) indicates significant differences between lactobacilli-treated and PBS controls at the basal time point; * (*p* < 0.05).

Lung macrophages play a key role in defense against pathogens; therefore, we aimed to study the ability of porcine *L. salivarius* strains to modulate the innate immune response of AM cultures when stimulated by poly(I:C) (Figure 3) or *S. pneumoniae* (Figure 4). For this set of experiments, the AMs of mice receiving *L. salivarius* FFIG58 (positive strain) and FFIG79 (negative strain) were cultured and challenged in vitro with poly(I:C) or the respiratory pathogen. AMs obtained from mice nasally primed with the FFIG58 strain showed increased basal levels of IFN-β, IFN-γ, IL-6, IL-10, IL-12, and IL-27. This effect was not achieved by the FFIG79 strain (Figure 3 and Figure 4).

The stimulation with poly(I:C) increased the production of IFN-β, IFN-γ, IL-6, IL-10, IL-12, and IL-27 in the AMs obtained from FFIG58-treated and control groups (Figure 3). However, the AM cultures from the FFIG58 group had significantly higher levels of IFN-β, IFN-γ, IL-6, and IL-27 than the controls. A similar tendency was observed when studying the response of AMs to the challenge with *S. pneumoniae* (Figure 4). The pneumococcal challenge increased the levels of all the studied cytokines when compared to basal levels. AMs obtained from FFIG58-treated mice showed increased levels of IFN-β, IFN-γ IL-6, IL-10, and IL-27 in comparison with the controls. In contrast, FFIG79 did not show any ability to differentially modulate the production of these cytokines in the presence of poly(I:C) (Figure 3) or *S. pneumoniae* (Figure 4), compared to the control group.

### 2.4. Long-Term Protection Conferred by Porcine L. salivarius FFIG58 against Secondary Pneumococcal Infection

**Figure 5:**
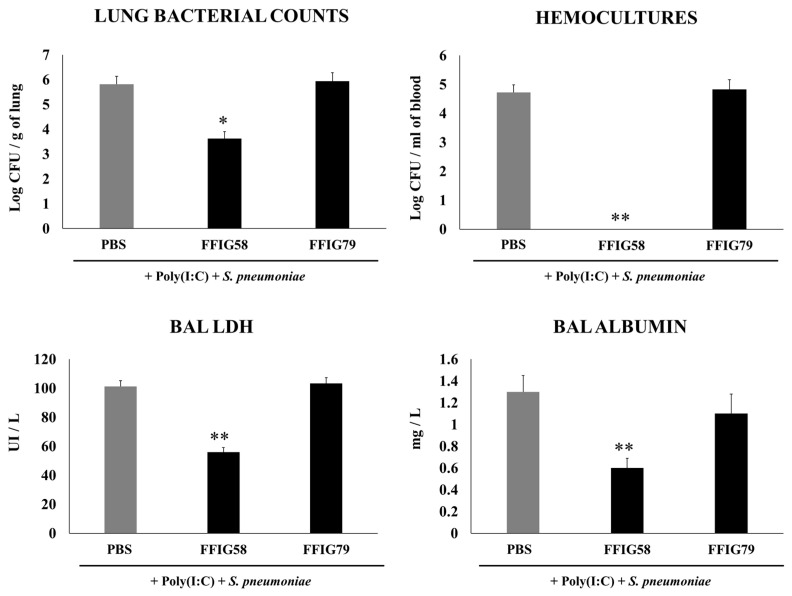
Effect of *L. salivarius* FFIG58 (positive strain) and FFIG79 (negative strain) on the long-term resistance to pneumococcal pneumonia. Infant mice were nasally primed with FFIG58 or FFIG79 during two consecutive days, then stimulated with three once-daily doses of poly(I:C) and, finally, challenged with *S. pneumoniae* serotype 6B thirty days after the last administration of poly(I:C). Infant mice treated with PBS (vehicle), stimulated with poly(I:C), and then challenged with *S. pneumoniae* were used as controls. Lung bacterial cells counts, hemocultures, lactate dehydrogenase (LDH) activity, and albumin concentrations in bronchoalveolar lavages (BALs) were determined on day 2 post-pneumococcal-challenge. Asterisks indicate significant differences between lactobacilli-treated and control groups; * (*p* < 0.05) and ** (*p* < 0.01).

Considering the immunomodulatory and protective abilities of *L. salivarius* FFIG58 in the respiratory tract, we wondered if this strain would be capable of generating long-lasting effects. To test this hypothesis, mice were nasally primed with strains FFIG58 and FFIG79 and stimulated with poly(I:C), as described previously. Animals were challenged with *S. pneumoniae* 30 days after the last poly(I:C) administration. Two days after the pneumococcal challenge, the resistance to the infection was evaluated by the determination of lung and blood bacterial cell counts, and BAL albumin content and LDH activity (Figure 5).

The nasal priming with the FFIG58 strain induced a significant reduction in lung bacterial counts, while no colonies were detected in the hemocultures (Figure 5). Moreover, the treatment with *L. salivarius* FFIG58 was effective in diminishing lung tissue damage, as shown by the reduction in the levels of albumin content and LDH activity. As expected, the non-immunomodulatory strain FFIG79 had *S. pneumoniae* counts in lung and blood as well as BAL albumin and LDH that were comparable to the control group (Figure 5).

### 2.5. Long-Term Effects of L. salivarius FFIG58 on Alveolar Macrophages in Response to Secondary Pneumococcal Infection

**Figure 6:**
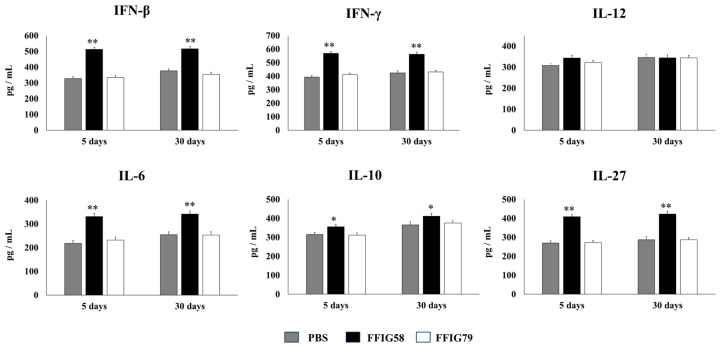
Effect of *L. salivarius* FFIG58 (positive strain) and FFIG79 (negative strain) on the long-term modulation of the levels of interferons (IFN)-β and IFN-γ, and interleukins (IL)-6, IL-10, IL-12, and IL-27 in pneumococcal pneumonia. Infant mice were nasally primed with FFIG58 and FFIG79 during two consecutive days, then stimulated with three once-daily doses of poly(I:C) and finally challenged with *S. pneumoniae* serotype 6B five and thirty days after the last administration of poly(I:C). Infant mice treated with PBS (vehicle), stimulated with poly(I:C), and then challenged with *S. pneumoniae* were used as controls. The levels of IFN-β and IFN-γ and interleukins IL-6, IL-10, IL-12, and IL-27 in bronchoalveolar lavages (BALs) were determined on day 2 post-pneumococcal-challenge. Asterisks indicate significant differences between lactobacilli-treated and control groups; * (*p* < 0.05) and ** (*p* < 0.01).

**Figure 7:**
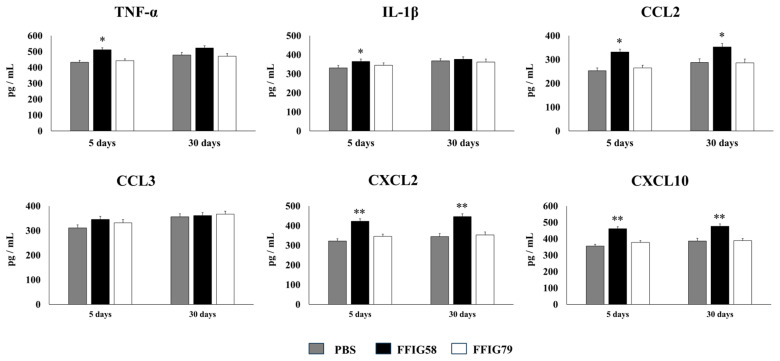
Effect of *L. salivarius* FFIG58 (positive strain) and FFIG79 (negative strain) on the long-term modulation of the levels of tumor necrosis factor (TNF)-α, interleukin (IL)-1β, chemokine C-C motif ligand 2 (CCL2), chemokine C-C motif ligand 3 (CCL3), chemokine C-X-C motif ligand 2 (CXCL2), and chemokine C-X-C motif ligand 10 (CXCL10) in pneumococcal pneumonia. Infant mice were nasally primed with FFIG58 or FFIG79 during two consecutive days, then stimulated with three once-daily doses of poly(I:C) and finally challenged with *S. pneumoniae* serotype 6B five and thirty days after the last administration of poly(I:C). Infant mice treated with PBS (vehicle), stimulated with poly(I:C), and then challenged with *S. pneumoniae* were used as controls. The levels of TNF-α, IL-1β, CCL2, CCL3, CXCL2, and CXCL10 in bronchoalveolar lavages (BALs) were determined on day 2 post-pneumococcal-challenge. Asterisks indicate significant differences between lactobacilli-treated and control groups; * (*p* < 0.05) and ** (*p* < 0.01).

In order to evaluate the long-term effects of the strain *L. salivarius* FFIG58 on AMs, mice were nasally primed with strains FFIG58 and FFIG79 and stimulated with poly(I:C). AMs were isolated from mice 5 or 30 days after the last poly(I:C) administration and stimulated in vitro with *S. pneumoniae*. The production of IFN-β, IFN-γ, IL-6, IL-12, IL-10, and IL-27 (Figure 6), and TNF-α, IL-1β, CCL2, CCL3, CXCL2, and CXCL10 (Figure 7) was evaluated in culture supernatants of AMs, taking into account that these cytokines are differentially regulated in AMs with a trained immunity phenotype [17].

AMs obtained from animals that received *L. salivarius* FFIG58 for 5 or 30 days before the pneumococcal challenge produced significantly higher levels of IFN-β, IFN-γ, and IL-6, the regulatory cytokines IL-10 and IL-27 (Figure 6), and the chemokines CCL2, CXCL2, and CXCL10 (Figure 7) than their respective controls. The FFIG58 strain also increased the production of TNF-α, and IL-1β (Figure 7) after 5 days, but the effect was not maintained over time, since the production of these cytokines in AMs obtained from *L. salivarius* FFIG58-treated mice 30 days before the pneumococcal challenge were similar to those of the controls. In addition, the production of IL-12 (Figure 6) and the proinflammatory chemokine CCL3 (Figure 7) in AMs from FFIG58-treated animals was not significant different than controls in both time points. As expected, the stimulation of mice with *L. salivarius* FFIG79 did not produce changes in the levels of any of the studied cytokines in any time point.

## 3. Discussion

Immunobiotics are able to beneficially modulate the immune system and enhance resistance against pathogens [13,14,15,16,17,18]. In previous studies, we isolated strains of the species *L. salivarius* from the intestine of wakame-fed pigs [13], and characterized them according to their abilities to modulate the innate immune response of intestinal epithelial cells after the activation of TLR3 and TLR4 signaling pathways [14]. We observed that the immunomodulatory properties were strain-specific; the FFIG58 and FFIG35 strains standing out as those with the greatest probiotic potential [14]. Moreover, experiments carried out in a model of enterotoxigenic *E. coli* (ETEC)/rotavirus superinfection in intestinal epithelial cells demonstrated the capacity of both FFIG35 and FFIG58 strains to differentially modulate the expression of IFN-β, IFN-λ, and antiviral factors, and to reduce rotavirus replication [19]. In addition, in vivo studies in mice showed the ability of the porcine lactobacilli to modulate the innate immune response and protect against ETEC infection [19]. Considering these results, we evaluated if porcine *L. salivarius* strains, when nasally administered, could be used to favorably stimulate the respiratory tract immunity in order to confer protection against infectious pathogens. We demonstrated that the nasal priming of mice with *L. salivarius* FFIG58 beneficially modulates respiratory immunity and confers long-term protection against *S. pneumoniae*. The results presented here allowed us to arrive to four key conclusions, as described below.

(a) *Immunobiotic strains can exert immunomodulatory properties in a different host and/or mucosal tissue from which they were isolated.* The classic search for probiotics that can exert beneficial effects on mucosal tissues like the gastrointestinal, respiratory, or urogenital tracts is based on the isolation and characterization of strains taken from the same ecological niche in which the microorganism will be applied. This type of strategy is based on the concept that microorganisms from a certain niche have the necessary adaptations to colonize and interact positively with the host. However, there is considerable evidence that foreign microorganisms, isolated from different mucous membranes, foods, and even different species, can exert immunomodulatory activities beneficial to the host. An example of this phenomenon is the demonstrated ability of the probiotic strain *Lacticaseibacillus rhamnosus* CRL1505, originally isolated from goat milk, to modulate intestinal and respiratory immunity when administered orally [16] or nasally [17], respectively. Similarly, the well-characterized probiotic strain *L. rhamnosus* GG, isolated from the intestine, is able to modulate respiratory immunity when nasally administered [18]. In line with these studies, we showed, in this work, that the strain FFIG58 from porcine intestine is capable of modulating immunity in the respiratory tract of mice. In this way, strains with remarkable immunomodulatory properties and the ability to protect against infections in a specific mucosa could be evaluated in other mucosal tissues, to establish if they can also exert protective effects. Furthermore, considering that mice are often used as preclinical models for the application of probiotics for the improvement of human health, it would be interesting to evaluate whether *L. salivarius* FFIG58 can exert beneficial effects in the context of human respiratory infections. This is an interesting topic for future research.
(b) *Porcine L. salivarius strains differentially modulate the respiratory innate immune response in a strain-dependent manner.* Using a mouse in vivo model of respiratory superinfection in which *S. pneumoniae* infects animals after the stimulation with poly(I:C) [15,20,21], we demonstrated that the immunomodulatory properties of the porcine *L. salivarius* strains and their potential to confer protection in the respiratory tract was a strain-specific characteristic. In our hands, the FFIG35 and FFIG58 strains had the ability to modulate respiratory immunity, while *L. salivarius* FFIG79 did not exert immunomodulatory effects, in accordance with the results obtained in intestinal epithelial cells [14]. These strains decreased lung and blood pneumococcal counts and reduced lung tissue damage evidenced by lower albumin levels and LDH activity. The nasal treatment of infant mice with *L. salivarius* FFIG35 or FFIG58 improved the production of IFN-β, IFN-γ, and IL-6 in the respiratory tract, factors that are critical to conferring protection against RSV [22] and pneumococci [23,24]. In this regard, it is well established that pneumococcal infections are more frequent and severe in the elderly. It was demonstrated that the production of IFN-β during *S. pneumoniae* infection was decreased in aged hosts compared to immunocompetent adults, and was associated with a reduced clearance of the pathogen [25]. Furthermore, this work demonstrated that despite similar levels of phagocytosis when compared to young hosts, aged macrophages produced significantly less IFN-β in response to pneumococcal infection. It was also shown that pneumococci defective in the virulence factor autolysin LytA were more efficiently phagocyted by macrophages than wild-type bacteria, leading to higher levels of cytosolic pneumococcal DNA accumulation, and higher expression of IFN-β, interferon-stimulated genes, TNF-α, and IL-1β, which promoted an improved clearance of *S. pneumoniae* [26]. Similarly, IFN-γ mediated protective effects against pneumococci, as mice deficient in this immune factor had impaired bacterial clearance [27,28]. On the other hand, it was shown that the impairment of IL-6 production in primary human monocytes cultures by influenza virus infection significantly increased their susceptibility to *S. pneumoniae* infection [29]. In line with those results, in vivo studies in IL-6^−/−^ mice showed that the influenza–pneumococci co-infection was characterized by enhanced bacterial burden and dissemination as well as aggravated pulmonary lesions that correlated with high mortality in comparison with wild-type animals [30]. The work demonstrated that the protective effect of IL-6 was associated with appropriate macrophage function, since this cytokine has a key role in macrophage death and their capacity to control lung tissue inflammation [31].
(c) *Alveolar macrophages are a key respiratory immune cell population involved in the beneficial effects induced by L. salivarius FFIG58.* AMs are the most abundant immune cell in the lungs, playing a critical role in homeostasis, host defense, and tissue remodeling [32]. Their phagocytic activity is crucial for the elimination of pathogens and infected cells during the course of infections. AMs also produce cytokines and chemokines that act on surrounding immune and epithelial cells inducing the transcription of immune factors that favors pathogens clearance [33]. Our previous studies demonstrated a key role of AMs in the beneficial effects induced in respiratory immunity by nasally administered probiotics [17,34]. In line with those previous studies, we observed, here, that *L. salivarius* FFIG58 improved the production of IFN-β, IFN-γ, and IL-6 in AMs in response to both poly(I:C) and *S. pneumoniae* challenges. The results indicate that AMs have a relevant role in the protective effect of the FFIG58 strain in the context of respiratory superinfection.

In addition to the improved production of inflammatory factors by AMs from FFIG58-treated mice, we also observed that the nasal treatment of mice with *L. salivarius* FFIG58 significantly increased the levels of the regulatory cytokine IL-10 in the respiratory tract as well as the production of IL-10 and IL-27 by AMs, immune factors that are involved in the protection of lung against the inflammatory damage [15,35]. A correct balance between pro- and anti-inflammatory cytokines is necessary to finely regulate the immune response, avoiding aberrant reactions and minimizing tissue damage [35]. During pneumococcal infection in mice, IL-10 production is required to temper an excessive lung injury and to improve survival [36,37], while IL-27 in combination with IL-6 are involved in the ability of AMs to ty to promote Treg cell responses [38]. These data indicate the important role of AMs in the regulation of inflammatory responses in the respiratory tract and highlight the capacity of the FFIG58 to improve protection of the lung against inflammatory-mediated damage through the modulation of AMs.

(d) *The generation of “trained” alveolar macrophages would be involved in the beneficial effects induced by L. salivarius FFIG58 in the respiratory tract.* Interestingly, we demonstrated, here, that the nasal priming of mice with *L. salivarius* FFIG58 can confer long-term protection against *S. pneumoniae*. Research over the last decade has shown that, after a first stimulus with an antigen, AMs can develop an innate immune memory phenotype [12,39,40,41]. This response, called “trained immunity”, stands out for its greater speed and enhanced capacity to produce cytokines after a second stimulus with the same or non-related antigen [12,40]. It has also been shown that the production of IFN-γ is essential for the generation of trained immunity in AMs [39]. Thus, we aimed to evaluate the ability of *L. salivarius* FFIG58 to generate trained immunity in mice, evaluating the protection against secondary pneumococcal pneumonia over the time. It was observed that the protective effect of the FFIG58 strain against secondary *S. pneumoniae* infection persisted for at least one month. Furthermore, the production of cytokines of AMs obtained from animals treated with FFIG58 and challenged with pneumococci after 5 and 30 days was comparable, demonstrating that the beneficial effects were maintained during this time. AMs from FFIG58-treated animals were capable of producing higher levels of IFN-β, IFN-γ, IL-6, IL-10, and IL-27 than controls after 5 and 30 days, which are important factors in the defense against pneumococci, as described previously. Furthermore, AMs produced higher levels of the inflammatory chemokines CCL2 (MCP-1), CXCL2 (MIP2-α), and CXCL10 (IP-10), both at 5 and at 30 days.

The kinetics of chemokine production is important for infection control in the lung since these immune factors are responsible for generating the migration of immune cells to the site of infection. The chemokines CCL2, CXCL2, and CXCL10 have been shown to be involved in the protection against *S. pneumoniae* in the respiratory tract. CCL2 recruits monocytes and macrophages [42]. It was reported that the oral administration of EM900, a non-antibiotic macrolide with an immunomodulatory effect, improved the production of CCL2 by macrophages in mice infected with *S. pneumoniae* [43]. This effect was associated with reduced pneumococcal counts in the respiratory tract and a diminished mortality of animals with invasive pneumococcal disease. In addition, it was shown that mice deficient in the macrophage migration inhibitory factor (MIF^−/−^) are highly susceptible to *S. pneumoniae* infection [44]. The deficiency of MIF was associated with reduced numbers of macrophages, as well as an impaired upregulation of CCL2 in the respiratory tract, highlighting the role of this immune mechanism in the protection against pneumococcal infection. It was also shown that infant mice (7 days old) are less efficient than adult animals (6 weeks old) in clearing the colonization of different pneumococcal serotypes, and that this phenomenon is related to a slower macrophage recruitment associated with an impaired ability to upregulate CCL2 [45]. On the other hand, CXCL2 acts as chemoattractant on neutrophils [46], which are critical for bacterial clearance and host survival in *S. pneumoniae*-challenged mice [47]. One study reported that IL-12 administration can improve innate defenses against pneumococcal infection by inducing IFN-γ and CXCL2 production, and thereby increasing the recruitment and activation of neutrophils [27]. Additionally, CXCL10 attracts lymphocytes, monocytes, and natural killer cells [48] and exhibits direct antimicrobial activity against *S. pneumoniae* [49]. Given that the ability to produce cytokines and chemokines more efficiently in response to a second stimulus is a characteristic attributed to AMs that have acquired a trained immunity phenotype, our results allow us to affirm that the FFIG58 strain would induce trained immunity in the respiratory tract. Future research will be focused in exploring the metabolic and epigenetic changes in AMs induced by the treatment with *L. salivarius* FFIG58 to establish, more precisely, the impact of this strain on the trained immunity of the respiratory tract and its long-term protection against pneumococcal infection.

## 4. Materials and Methods

### 4.1. Strains and Culture Conditions

*L. salivarius* FFIG35, FFIG56, FFIG58, FFIG79, and FFIG124 were isolated from the small intestine of wakame-fed pigs [13]. *L. salivarius* strains were cultured for 16 h at 37 °C (final log phase) in Man–Rogosa–Sharpe broth (MRS, Oxoid, Hampshire, UK). The bacteria were harvested by centrifugation at 4000× *g* for 10 min, washed two times with sterile 0.01-mol/L phosphate-buffered saline (PBS, pH 7.2), and resuspended in sterile PBS before use.

### 4.2. Animals and Treatments

Infant (3-week-old) BALB/c mice were provided by the closed colony of CERELA (San Miguel de Tucumán, Argentina). Animals were housed in plastic cages at room temperature and kept in strict accordance with the recommendations in the Guide for the Care and Use of Laboratory Animals of the Guidelines for Animal Experimentation of CERELA. The assays for each parameter studied were performed with 5–6 mice per group. *L. salivarius* strains were nasally administered to infant mice for 2 consecutive days at a dose of 10^8^ cells/mouse/day in 50 µL of PBS [15,21]. Animals receiving only PBS (vehicle) were used as controls. Mice were fed with a conventional balanced diet ad libitum. The CERELA Institutional Animal Care and Use Committee prospectively approved this research under the protocol BIOT-CRL-18. All efforts were made to minimize the number of animals and their suffering. No signs of discomfort or pain were observed before mice reached the endpoints. No deaths were observed before mice reached the endpoints.

### 4.3. Poly(I:C) Administration and Respiratory Infections

Administration of the TLR3 agonist poly(I:C) was performed 2 days after the last day of treatment with *L. salivarius* strains or PBS (control group). Mice were stimulated with 100 µL of PBS containing 250 µg of poly(I:C) (equivalent to 10 mg/kg body weight), which was administered dropwise via the nares [15,20,21]. Mice received 3 doses of poly(I:C) with a 24 h rest period between each administration. *S. pneumoniae* serotype 6B was grown on blood agar for 18 h. Colonies were suspended in Todd Hewitt broth (Oxoid, Hampshire, UK), incubated overnight at 37 °C, harvested, and washed with sterile PBS. Cell density was adjusted to 4 × 10^7^ CFU/mL [15]. Challenge with pneumococci was performed 5 or 30 days after the last administration of poly(I:C) and mice were sacrificed 2 days after the challenge. Lungs were extracted, weighed, and homogenized in sterile peptone water. Then, lung samples were diluted appropriately, plated in duplicate on blood agar, and incubated for 18 h at 37 °C. *S. pneumoniae* counts were expressed as log of CFU/g of a lung or CFU/mL of blood.

### 4.4. Lung Injury Parameters

Bronchoalveolar lavage (BAL) samples were obtained, as described previously [15]. Briefly, a small incision was made with the scalpel in order to expose the trachea and then a cannulation was performed with a catheter. After that, 2 sequential lavages with sterile PBS were performed in each mouse. The recovered BAL fluid was centrifuged for 10 min at 900× *g* and frozen at −70 °C until analysis. Albumin content and lactate dehydrogenase (LDH) activity were determined in the acellular BAL fluid, as described previously [15].

### 4.5. Alveolar Macrophage Primary Cultures

Alveolar macrophage primary cultures were carried out in accordance with previous publications [50,51]. Murine AMs were obtained from infant mice by performing bronchoalveolar lavage. The obtained fluids were transferred to new sterile tubes, washed twice in sterile PBS, and resuspended in RPMI 1640 medium with 10% FBS, 1 mM L-glutamine, and 100 U/mL penicillin–streptomycin. BAL cells were seeded in 24-well plates and incubated for 2 h at 37 °C in 5% CO_2_ to promote adherence. Non-adherent cells were washed, and macrophages were maintained for 24 h before stimulation. AMs were stimulated with *S. pneumoniae* (multiplicity of infection (MOI) of 3). The supernatant of the cultures was extracted 12 h after the exposure to pneumococci for the determination of the secreted cytokines.

### 4.6. Cytokine Concentrations in BAL and Culture Supernatants

Cytokine concentrations in BAL and culture supernatants samples were measured following the manufacturer’s recommendations (R&D Systems, Minneapolis, MN, USA) using the following enzyme-linked immunosorbent assay (ELISA) kits: Mouse IFN-beta enzyme-linked immunosorbent assay (ELISA) Kit, Mouse IFN-gamma Quantikine ELISA Kit, Mouse IL-6 Quantikine ELISA Kit, Mouse IL-10 Quantikine ELISA Kit, Mouse IL-12 p70 DuoSet ELISA, and Mouse IL-27 p28/IL-30 Quantikine ELISA Kit. CCL2 (Mouse MCP1 ELISA Kit (ab208979)), CCL3 (Mouse MIP1a ELISA Kit (ab200017)), CXCL2 (Mouse MIP2 ELISA Kit (ab204517)), and CXCL10 (Mouse IP-10 ELISA Kit (ab214563)) were measured with commercially available ELISA kits following the manufacturer’s recommendations (Abcam, Cambridge, UK).

### 4.7. Statistical Analysis

The experiments were performed in triplicate and the results were expressed as mean ± SD. Statistical analyses were performed using Prism 8.0 (GraphPad software). Comparisons among multiple groups across multiple time points were performed using a two-way ANOVA with Tukey’s multiple comparison post hoc test. Comparisons between two groups were performed using unpaired Student’s t-tests. Differences were considered significant at *p* < 0.05.

## 5. Conclusions

In this work we characterized the capacity of different *L. salivarius* strains, originally isolated form the porcine intestine, to regulate the immune response to infections in the respiratory tract. We demonstrated that nasal priming with *L. salivarius* FFIG58 can improve protection against a secondary pneumococcal infection, with an effect that lasts up to 30 days. The strain FFIG58 was able to modulate AM function and the results suggest that the treatment would promote the development of trained immunity in the respiratory tract. The nasal administration of *L. salivarius* FFIG58 could constitute a viable strategy to modulate innate immunity in the human or porcine host, conferring protection against bacterial and viral pathogens. Future work will focus on studying the interaction of the FFIG58 strain with human or porcine alveolar macrophages and respiratory epithelial cells, as well as confirming its ability to modulate respiratory immunity in in vivo experiments in pigs or clinical trials in humans.

## Data Availability

The data presented in this study are available throughout the article.
